# Integrating Cranio-Cervical Neuromyofascial Continuity With Body-Wide Function in Kimmerle Anomaly: An Osteopathic Case Report

**DOI:** 10.7759/cureus.98352

**Published:** 2025-12-02

**Authors:** Gianfranco Pizzolorusso, Vito Borraccia, Cosimo Quaranta, Giandomenico D'Alessandro, Christian Lunghi

**Affiliations:** 1 Research Department, A.T. Still Academy, Grottaglie, ITA; 2 Research Department, Academy of Osteopathy and Anatomy, Chieti, ITA; 3 Research Department, BMS Formation, Paris, FRA; 4 Clinical-Based Human Research Department, Foundation Centre for Osteopathic Medicine (COME) Collaboration, Pescara, ITA

**Keywords:** arcuate foramen, cervical atlas, osteopathic care, osteopathic medicine, ponticulus posterior, somatic dysfunction, tertiary prevention

## Abstract

Kimmerle anomaly is a congenital bony variant of the atlas vertebra that can, in rare cases, be associated with neurological and vascular symptoms. Although often asymptomatic, its clinical significance increases when presenting alongside persistent cranio-cervical pain and associated symptoms. This case report aims to describe the clinical presentation and osteopathic management of a young adult with symptoms potentially secondary to Kimmerle anomaly, highlighting the role of osteopathic manipulative treatment in a complex diagnostic context. A 19-year-old male presented with chronic cranio-cervical pain, right-sided neck and trapezius discomfort, tension-type headaches, dizziness, and episodic right-ear tinnitus and hypoacusis. Symptoms began at the age of 16 years and persisted despite various conservative interventions. Imaging revealed a complete Kimmerle anomaly. Neurological examination by the osteopath indicated altered sensation and sensory deficits. The patient underwent five osteopathic manipulative treatment sessions using a whole-body approach, focused on somatic dysfunctions, including, but not limited to, the upper cervical spine and its related fascial system connections. Techniques were chosen based on clinical evaluation and adapted to the patient’s individual dysfunction patterns. No adverse effects were reported. At follow-up, the patient reported a reduction in pain, improved quality of life, and resolution of vestibular and auditory symptoms. This case highlights the potential value of osteopathic assessment and treatment in patients with persistent symptoms potentially linked to Kimmerle anomaly. The integration of osteopathic care into interdisciplinary diagnostic pathways may enhance management and improve outcomes in complex cases where structural anomalies coexist with functional disturbances.

## Introduction

Anatomic variations of the first cervical vertebra have been extensively documented in the literature, including the Kimmerle anomaly (KA) [1], also referred to as the ponticulus posterior (PP) or the arcuate foramen of the atlas [2]. PP is an anatomical anomaly involving a bony bridge over the vertebral artery (VA) at the posterior arch of the atlas, which can have clinical implications, particularly during surgeries involving the upper cervical spine. A study by Sekerci et al. (2015) [3] using cervical 3D cone-beam computed tomography (CBCT) found a prevalence of 36.8% in a Turkish population, with bilateral complete PP observed in 6.3% and partial PP in 16.2%. The prevalence varied between males and females, but no significant difference was found across age groups. This study highlighted the relevance of PP in clinical procedures on the upper cervical spine, as it may pose risks during interventions such as C1 lateral mass screw insertion. In a broader meta-analysis, Elliott and Tanweer (2014) [4] reviewed 44 studies, including 21,789 patients, and found a prevalence of 16.7% for PP across radiographic and cadaveric studies. The anomaly was bilaterally present in 5.4% of cases, and its presence was more common in cadaveric studies (18.8%) than in CT scans (17.2%). They also identified that PP may give the false impression that the posterior arch of the atlas is sufficiently robust for C1 lateral mass screw insertion, which could increase the risk of VA injury during surgery. A meta-analysis by Pekala et al. (2017) [5] of 127 studies (55,985 subjects) estimated the pooled prevalence of a complete foramen arcuale (FA) at 9.1%, with incomplete FA at 13.6%. The FA was more common in North Americans and Europeans, and it posed potential surgical risks, especially during neurosurgery of the atlas. The study suggested preoperative screening with CT to detect the presence of FA. A study by Gibelli et al. (2016)[6] examined the prevalence of PP in a Northern Italian orthodontic sample of 221 patients, revealing a 7.7% prevalence for complete PP and 9.0% for incomplete PP. No significant differences were observed between genders. This study provided the first data on the prevalence of PP in Italy, suggesting the need for further research, especially using advanced imaging techniques such as CT and CBCT scans to assess the prevalence in different populations. Overall, the prevalence of ponticulus posticus varies across populations, with significant implications for clinical practice, particularly in procedures involving the upper cervical spine. Preoperative imaging, such as CT or CBCT, is essential for identifying this anatomical variation to avoid complications during surgery.

Kimmerle anomaly: anatomical features and clinical implications

In patients with KA, the posterior atlanto-occipital membrane forms a bony bridge, known as the posterior ponticle, between the posterior arch of C1 and the lateral mass [2]. The resulting bony bridge, referred to as the PP, narrows the VA, which is surrounded by the venous plexus and branches of the sympathetic plexus [1]. Additionally, the suboccipital nerve, also known as the dorsal ramus of the first cervical nerve, is responsible for motor innervation of the suboccipital muscles [2]. More specifically, after ascending the transverse foramen of C1, the VA turns posteriorly and medially around the superior articular process of C1 [2]. It does not enter the cranium but instead passes beneath the opening of the posterior atlanto-occipital membrane [2].

Furthermore, the suboccipital nerve emerges superior to the posterior arch and inferior to the VA, innervating the rectus capitis posterior major and minor, the obliquus capitis superior and inferior, and the semispinalis capitis muscles [2]. Besides congenital and genetic-trait hypotheses, some studies have also suggested a role for aging and external mechanical forces, including intentional cranial deformation [1]. Given the anatomical structures described above, the presence of a PP can have significant clinical implications, including neurological conditions resulting from compression of the neurovascular bundle at the level of the atlas [2]. Clinical manifestations may result from compression or stretching of the VA and its venous and neural plexuses at the cranio-cervical junction. Symptoms of Barre-Lieou syndrome include headache, retro-orbital pain, vasomotor disturbances of the face, and recurrent disturbances of vision, swallowing, and phonation [1]. Additional manifestations may include dizziness, nausea, vomiting, disturbances of equilibrium and visuo-auditory function, orofacial pain, loss of consciousness, acroparesthesia, and vegetative symptoms [1]. These symptoms have also been linked to structural and functional alterations of the suboccipital muscles and their fascial interconnections [7].

Overview of treatment options

The first treatment option for patients presenting with clinical signs and symptoms of KA should be conservative: surgical treatment of VA compression in patients with KA is preferable in cases where conservative treatment is inefficient [8]. In a clinical report by Kuć et al. [5], soft tissue mobilization was found to be effective in treating a single patient with temporomandibular disorders and symptoms secondary to KA. In contrast, a study by Lvov et al. [8] indicated that conservative treatments, such as physiotherapy, were not successful, and surgery emerged as the most effective option for managing KA-related symptoms.

Several surgical techniques have been described in the literature to address vertebral-vascular compression at the level of C1. Clinical results in treating patients undergoing surgery have also been reported, documenting an overall improvement in symptoms [8]. Finally, a case report on soft tissue mobilization in a patient with temporomandibular joint disorder and KA documented both subjective and objective outcomes [9].

Aim of the case report

To our knowledge, the effects of osteopathic manipulative treatment (OMT) in patients with KA have never been documented. The present case report aims to document the application and outcomes of OMT in a single patient diagnosed with KA, who presented with cranio-cervical, neurological, and psychological symptoms. The CARE guidelines and checklist [10] were used to guide the presentation of this case report.

## Case presentation

Informed consent

All procedures performed in the present case report were in accordance with the ethical standards of the Ethics Committee of the Italian National Institute of Health (Comitato Etico Unico Regionale per la Basilicata - Azienda Ospedaliera Regionale “San Carlo” - Via Potito Petrone, 1 - 85100 Potenza. Prot. Segreteria TS/CEUR n. 20210023023 del 24.05.2021) and Helsinki Declaration. Written informed consent was obtained from the patient at the first visit, as well as permission for publication of this manuscript.

Patient information

A 19-year-old Italian man was referred to the practice of an osteopath who was trained in accordance with the osteopathic standard of practice informed by tradition and best available evidence [11] and who had completed one year of clinical practice. The osteopath explained to the patient the aim of the osteopathic evaluation and treatment. The patient signed the informed consent, provided a detailed clinical history, and explained his persistent symptoms. The patient’s chief complaint was diagnosed as a chronic tension-type headache by the family doctor. The nature of pain that the patient was experiencing is reported in Table 1, summarizing the Socrates Pain assessment [12].

**Table 1:** Socrates pain assessment. *: SOCRATES is a mnemonic acronym used by emergency medical services, physicians, nurses, and other healthcare professionals to evaluate the nature of pain that a patient is experiencing.

SOCRATES pain assessment*	
Site	Suboccipital region, radiating to the forehead, orbits, and periorbital area
Onset	Three years earlier
Character	Nociceptive, peripheral, nociplastic, with a secondary emotional component
Radiation	Radiating to the forehead, orbits, and periorbital area
Associations	Anxiety and depression
Time course	Constant from the day of the onset, and the patient could not identify any antalgic positions
Exacerbating/Relieving factors	The patient reported that any activity worsened the pain and that there were no relieving factors
Severity	The Visual Analog Scale score was 9 out of 10

The patient reported a recurrent pattern of pain during the day, with worsening throughout the night. At times, the headache was characterized by acute, intense, pulsating pain. The distribution of pain included the suboccipital area, radiating to the forehead, orbits, and periorbital region. Severe pain was also reported on the right side of the neck and trapezius, especially during rightward rotation of the head. Turning the head to the right also triggered dizziness and, at times, hearing loss (hypoacusia) and tinnitus in the right ear. The patient reported sharp, stabbing pain with intolerance to pressure from the spectacle arms and difficulty wearing headgear. Nonsteroidal anti-inflammatory drugs were ineffective. The patient described subjective cognitive clouding and a sense of disconnection, with impaired clarity of environmental perception and persistent imbalance. External observers noted facial swelling and partially closed eyelids. The patient also endorsed depressive symptoms attributed to the functional limitations imposed by the condition rather than to external stressors. Despite attempts to resume daily activities, the patient remained unable to do so and expressed a strong desire for recovery. Additional symptoms included sensations of floating, wooziness, and a feeling of head heaviness. Symptoms began three years earlier and gradually worsened over time, leading to deteriorating health, cessation of physical activities, and discontinuation of university studies. The patient denied any association between symptom onset and potential causes, including traumatic (e.g., head trauma, whiplash injury). In 2019, the patient consulted a family doctor, who referred him to a physiotherapist with a diagnosis of chronic neck pain. Anti-inflammatory drugs and 10 physical therapy sessions (including massage and instrumental therapy) did not result in any significant changes in the patient’s condition. The patient was later diagnosed with anxiety and depression, for which he declined pharmacological treatment and was referred for psychotherapy, which did not lead to symptom improvement. According to Fava et al. [13], the patient met all four criteria for allostatic overload, namely, (1) psychiatric symptoms, (2) psychosomatic symptoms, (3) significant impairment in social or occupational functioning, and (4) significant impairment in psychological well-being.

**Figure 1:**
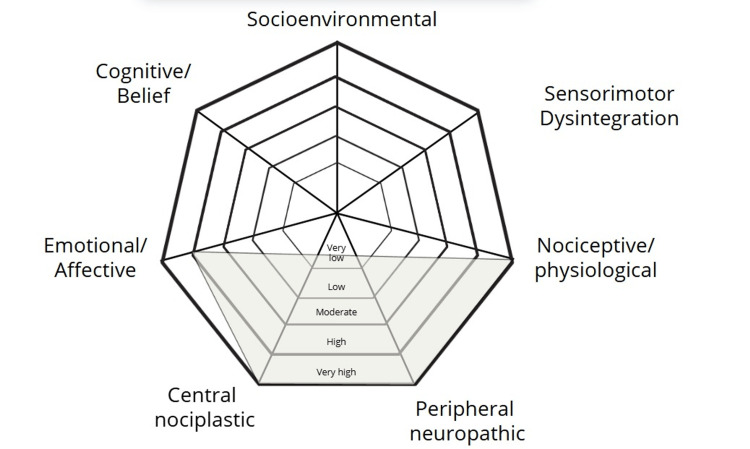
Pain assessment radar plot. The pain assessment radar plot shows the pathognomonic mechanism of pain onset, as well as the factors that contribute to the perception of pain.

The information emerging from the case history, the symptoms experienced by the patient (Table 1), previous treatment outcomes, and the physical examination (Figure 1) suggested the presence of nociceptive, peripheral, and nociplastic pain, with a secondary emotional and affective component [14].

Clinical findings

At the first osteopathic consultation, the patient did not appear anxious but seemed comfortable during both the clinical history and physical examination. The patient’s vital signs were within normal limits. The modified Romberg test of standing balance was positive [15]. Based on these clinical findings, the osteopath considered it appropriate to refer the patient to a medical specialist to further evaluate the positive test result, assess the potential proprioceptive rather than vestibular origin, and clarify the diagnosis. Physical examination revealed a limited range of motion and pain during neck extension testing. The pain was localized in the lower cervical spine, and neck extension elicited a right-sided pulsating headache, predominantly in the suboccipital region. Weakness of the right sternocleidomastoid was also noted during neck muscle strength testing. The patient described sensations from light touch with a cotton wisp as abnormally intense, unpleasant, or painful, and distinctly stronger than in other spinal segments, indicating sensory gain, allodynia, and hyperalgesia involving central pathways [16]. Based on the history and physical examination findings, the patient was referred to both an otolaryngologist and a neurologist.

Timeline

**Figure 2:**
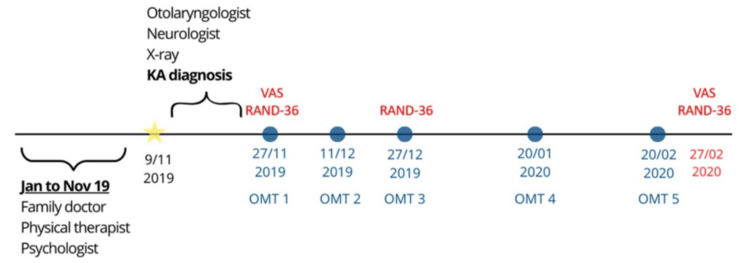
Timeline of main events. From January to November 2019, the patient followed the family doctor’s recommendations without improvement. The yellow star indicates the first osteopathic consultation, during which physical examination findings did not allow for OMT, and the patient was referred for further medical examinations. Each of the five blue circles represents an OMT session following the diagnosis of KA. Red marks indicate the days on which the RAND-36 questionnaire was completed by the patient. OMT: osteopathic manipulative treatment; KA: Kimmerle anomaly; RAND-36: RAND 36-Item Health Survey

The timeline of events, particularly those related to the main aspects of osteopathic care, is presented in Figure 2.

Medical diagnosis

**Figure 3:**
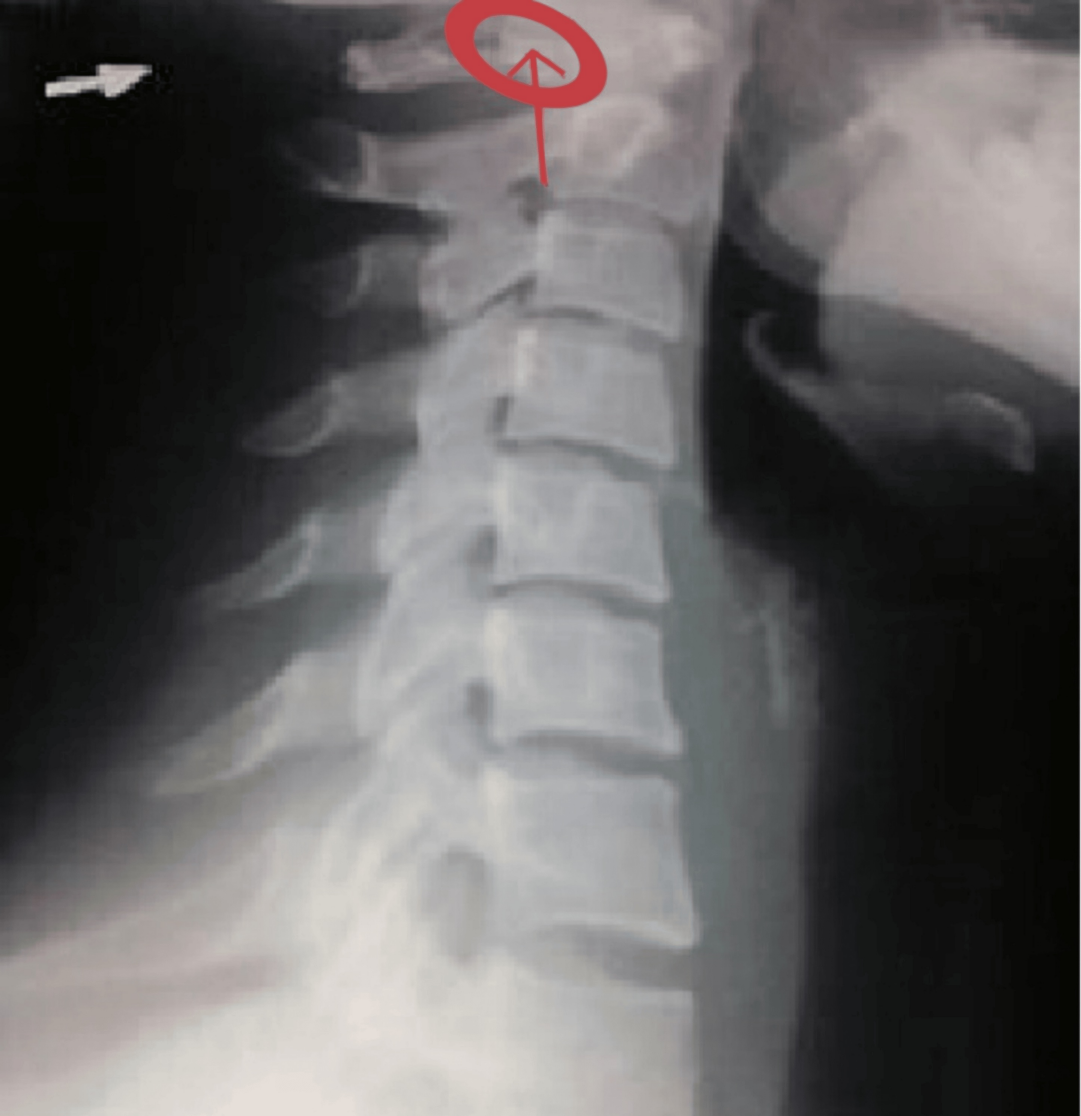
Cervical X-ray. Lateral cervical X-ray showing evidence of Kimmerle anomaly.

The otolaryngologist reported no vestibular deficit, as otoneurological findings were normal, including the absence of both spontaneous and positional nystagmus. Static posturographic assessment on a stabilometric platform revealed impairments in postural stability and sensory integration, suggesting a proprioceptive origin (i.e., cervicogenic dizziness) rather than a vestibular one. The neurologist conducted further investigations with a lateral cervical X-ray. The final diagnosis was KA, characterized by a complete and calcified bony ring on the right side of the atlas (Figure 3). According to both the otolaryngologist and the neurologist, the patient’s symptoms were attributable to KA. The patient was advised to continue OMT.

Therapeutic intervention: osteopathic care

Within osteopathic care, the osteopathic palpatory examination is central to clinical reasoning and OMT planning. Osteopathic palpatory examination was performed to assess for somatic dysfunction [17]. Somatic dysfunction is defined as “impaired or altered function of related components of the body framework system: skeletal, arthrodial, and myofascial structures, and their related vascular, lymphatic, and neural elements” [17]. The osteopathic palpatory examination aims to evaluate the whole body to identify palpatory findings related to low-grade inflammation, as indicated by tissue texture abnormalities, asymmetry, restriction of motion, and tenderness (the TART criteria) [17].

Consequently, somatic dysfunction is associated with 10 musculoskeletal body regions, classified according to the International Statistical Classification of Diseases and Related Health Problems (ICD) [17]. The inclusion of a code for somatic dysfunction within the ICD, specifically designated as M.99 in the ICD-10 version, is crucial for standardizing the clinical recognition and documentation of this related health problem, not a disease, but more of an illness state. This ensures consistency in osteopathic evaluation, treatment planning, and facilitates interprofessional communication and epidemiological research across diverse healthcare settings. The regions of the body framework coded in the ICD-10 include the head, cervical, thoracic, lumbar, sacral, pelvic, lower extremities, upper extremities, rib cage, abdomen, and others (the viscera fall into this category). The number of detected TART clinical signs is used to define the severity of somatic dysfunction as follows: none (0 signs), mild (1 sign), moderate (2 signs), or severe (3 or more signs) [18].

The osteopath aims to provide person-centered OMT based on clinically relevant somatic dysfunction [11]. The osteopath employs interoceptive touch by understanding how the physiological body functions, accommodating the patient’s affectivity through palpation focused on tissues guided by mental imagery of anatomical and physiological elements, and interpreting what the body is attempting to communicate [11]. This approach helps make sense of patients’ health processes and symptoms, allowing human bodies to be configured and enacted through touch-based osteopathic strategies [11], administered to regions of the somatic body associated with patient-altered functions (i.e., somatic dysfunction) [11].

Osteopathic encounter 1

The first osteopathic palpatory examination and OMT were scheduled one week after the neurologist’s diagnosis, with the patient presenting with KA on the right side of the atlas, as documented on the X-ray (Figure 3).

The patient was asked to complete the Visual Analog Scale (VAS) [19], where 0 and 10 indicate no pain and maximum pain, respectively. The patient’s VAS score was 9. The patient also completed the RAND 36-Item Health Survey, Version 1.0 (RAND-36) [20], validated in the Italian language [21]. It is important to note that both the SF-36 and RAND-36 scales are known for their high reliability in assessing health-related quality of life. This self-administered questionnaire consists of 36 items assessing health across the following eight dimensions: physical functioning, role limitations due to physical health, role limitations due to emotional problems, energy/fatigue, emotional well-being, social functioning, pain, and general health [20,21], with higher scores indicating a more favorable health status.

During the osteopathic palpatory examination, the patient exhibited an external health locus of control and demonstrated low body awareness. The physical examination and modified Romberg test of standing balance [15] showed the same findings as during the first osteopathic visit. Results from the osteopathic care session 1 (i.e., osteopathic palpatory examination and OMT) are presented in Table 2.

**Table 2:** Osteopathic diagnosis and treatment of the first osteopathic encounter. In the table, an “X” indicates that the reference element for the corresponding cell has been identified, while a “-“ denotes that the reference element has not been found. Body areas for the diagnosis and coding of somatic dysfunction are defined in the ICD-10-CM using codes M99.0-9 (where 0–9 represents the anatomical regions). OMT and OMTh procedures are coded for different body regions and techniques using the codes 7W00-09 (where 00-09 represents the anatomical regions) and X 0-9 Z (where 0–9 indicates the type of technique applied). ICD: International Classification of Diseases; OC: osteopathic care session; OPE: osteopathic palpatory examination; SD: somatic dysfunction; OR: other region, also used to indicate a generalized fascial pattern throughout the body; OMT: osteopathic manipulative treatment; OMTh: osteopathic manipulative therapy; PINS: progressive inhibition of neuromuscular structures technique; MET: muscle energy techniques; MFR: myofascial release; OCF: osteopathy in the cranial field

Osteopathic encounter 1									
OPE - SD								OMT	ICD
ICD	Region	Area	T	A	R	T	Severity	Technique	T. code
M99.00	Head	Occiput	x	-	-	x	Moderate	OCF	7W00X4Z
		Temporal m.	x	-	-	x	Moderate	PINS	7W00X9Z
		Masseter m.	x	-	-	x	Moderate	PINS	7W00X9Z
M99.01	Cervical	Suboccipital m.	x	-	-	x	Moderate	PINS	7W00X9Z
		C1	x	-	x	x	Severe	-	-
		Hyoid	x	-	-	-	Mild	-	-
		Supra-hyoid m.	x	-	-	x	Moderate	MET	7W01X7Z
		Infrahyoid m.	x	-	-	x	Moderate	MET	7W01X7Z
M99.02	Thorax	Clavicles	x	-	x	x	Severe	MFR	7W02X1Z
		T5-T6-T7	x	-	x	-	Moderate	-	-
		Diaphragm m.	x	x	x	x	Severe	MFR	7W09X1Z
M99.03	Lumbar	L5-S1	x	-	x	-	Moderate	-	-
M99.04	Sacral	Sacroiliac joints	x	x	x	x	Severe	MFR	7W04X1Z

Based on the patient’s perception of local hypersensitivity and apprehension, the segmental dysfunction at the C1 level, despite presenting with the highest severity grade, was not treated. Similarly, the segmental dysfunctions at the thoracic (T5-T7) and lumbosacral (L5-S1) levels, which were graded as moderate in severity, were also not treated due to the patient’s limited tolerance and ongoing apprehension. The osteopathic care session 2 was scheduled two weeks after the first session.

Osteopathic encounter 2

At the osteopathic care session 2, the patient reported having experienced tinnitus for a couple of hours and diffuse muscular soreness lasting four to five hours after the first treatment. The patient did not use any medications or other medical remedies to alleviate the side effects he described.

The modified Romberg test of standing balance was negative, and the findings from the osteopathic palpatory examination were less severe than those observed during the osteopathic care session 1.

The osteopathic palpatory examination revealed nearly the same regions associated with SDs as in the previous session and showed an improvement in the severity of the segmental dysfunction at C1. The manual procedures used in the osteopathic care session 2 were in line with the rationale of the previous osteopathic care session (Table 3). A two-week interval was set between the second and third osteopathic care sessions.

**Table 3:** Osteopathic diagnosis and treatment of the second osteopathic encounter. In the table, an “X” indicates that the reference element for the corresponding cell has been identified, while a “-“ denotes that the reference element has not been found. Body areas for the diagnosis and coding of somatic dysfunction are defined in the ICD-10-CM using codes M99.0-9 (where 0–9 represents the anatomical regions). OMT and OMTh procedures are coded for different body regions and techniques using the codes 7W00-09 (where 00-09 represents the anatomical regions) and X 0-9 Z (where 0–9 indicates the type of technique applied). ICD: International Classification of Diseases; OC: osteopathic care session; OPE: osteopathic palpatory examination; SD: somatic dysfunction; OR: other region, also used to indicate a generalized fascial pattern throughout the body; OMT: osteopathic manipulative treatment; OMTh: osteopathic manipulative therapy; PINS: progressive inhibition of neuromuscular structures technique; MET: muscle energy techniques; MFR: myofascial release; OCF: osteopathy in the cranial field

Osteopathic encounter 2									
OPE - SD								OMT	ICD
ICD	Region	Area	T	A	R	T	Severity	Technique	T. code
M99.00	Head	Occiput	x	-	-	x	Moderate	OCF	7W00X4Z
		Temporal m.	x	-	-	x	Moderate	PINS	7W00X9Z
		Masseter m.	x	-	-	x	Moderate	PINS	7W00X9Z
M99.01	Cervical	Suboccipital m.	x	-	-	x	Moderate	PINS	7W01X9Z
		C1	x	-	-	x	Moderate	-	-
		Hyoid	x	-	x	-	Moderate	MFR	7W01X1Z
		Supra-hyoid m.	x	-	-	-	Mild	MET	7W01X7Z
		Infrahyoid m.	x	-	-	-	Mild	MET	7W01X7Z
M99.02	Thorax	Clavicles	x	-	x	-	Moderate	MFR	7W02X1Z
		T5-T6-T7	x	-	x	-	Moderate	-	-
		Diaphragm m.	x	x	x	-	Severe	MFR	7W02X1Z
M99.03	Lumbar	L5-S1	x	-	x	-	Moderate	-	-
M99.04	Sacral	Sacroiliac joints	x	x	x	-	Severe	MFR	7W04X1Z

Osteopathic encounter 3

**Figure 4:**
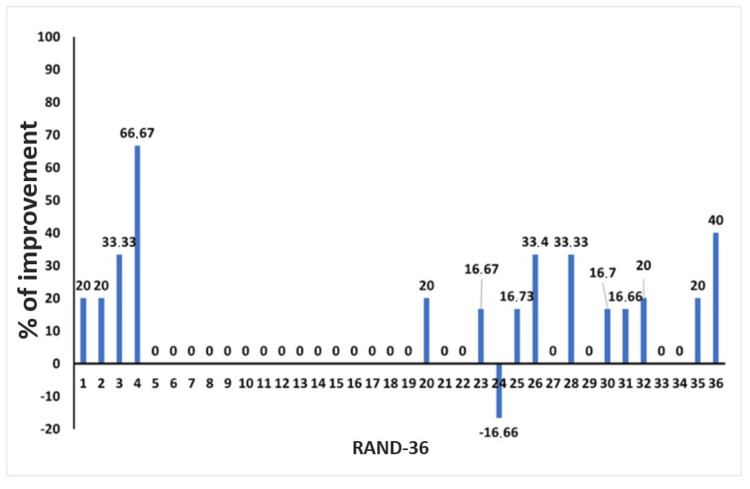
RAND-36 scores at the third osteopathic encounter. Percentage improvement in RAND-36 scores between November 27, 2019 and December 27, 2019. RAND-36: RAND 36-Item Health Survey

The patient reported no adverse effects following the second OMT. The modified Romberg test of standing balance was negative, and the osteopathic palpatory examination findings were even less pronounced than those observed during the osteopathic care session 2. The patient reported overall improvement in migraine, dizziness, and neck pain. The patient completed the RAND-36 (Figure 4), and the osteopathic palpatory examination revealed a significant reduction in SD severity (Table 4). The fourth osteopathic care session was scheduled after four weeks.

**Table 4:** Osteopathic diagnosis and treatment of the third osteopathic encounter. In the table, an “X” indicates that the reference element for the corresponding cell has been identified, while a “-“ denotes that the reference element has not been found. Body areas for the diagnosis and coding of somatic dysfunction are defined in the ICD-10-CM using codes M99.0-9 (where 0–9 represents the anatomical regions). OMT and OMTh procedures are coded for different body regions and techniques using the codes 7W00-09 (where 00-09 represents the anatomical regions) and X 0-9 Z (where 0–9 indicates the type of technique applied). ICD: International Classification of Diseases; OC: osteopathic care session; OPE: osteopathic palpatory examination; SD: somatic dysfunction; OR: other region, also used to indicate a generalized fascial pattern throughout the body; OMT: osteopathic manipulative treatment; OMTh: osteopathic manipulative therapy; PINS: progressive inhibition of neuromuscular structures technique; MET: muscle energy techniques; MFR: myofascial release; OCF: osteopathy in the cranial field

Osteopathic encounter 3									
OPE - SD								OMT	ICD
ICD	Region	Area	T	A	R	T	Severity	Technique	T. Code
M99.00	Head	Occiput	x	-	-	-	Mild	OCF	7W00X4Z
		Temporal m.	x	-	-	-	Mild	PINS	7W00X9Z
		Masseter m.	x	-	-	-	Mild	PINS	7W00X9Z
M99.01	Cervical	Suboccipital m.	x	-	-	-	Mild	PINS	7W01X9Z
		C1	x	-	-	-	Mild	-	-
		Hyoid	x	-	-	-	Mild	-	-
		Supra-hyoid m.	x	-	-	-	Mild	-	-
		Infrahyoid m.	x	-	-	-	Mild	-	-
M99.02	Thorax	Clavicles	x	-	-	-	Mild	-	-
		T5-T6-T7	x	-	x	-	Moderate	-	-
		Diaphragm m.	x	-	x	-	Moderate	MET	7W02X7Z
M99.03	Lumbar	L5-S1	x	-	x	-	Moderate	-	-
M99.04	Sacral	Sacroiliac joints	x	-	-	-	Mild	-	-

Osteopathic encounter 4

The patient reported no adverse events after the third OMT and experienced no dizziness, although he had two episodes of mild cervicogenic headache that did not require medication. Furthermore, during the previous week, he started a diet and practiced yoga twice. The osteopathic palpatory examination revealed somatic dysfunctions associated with the head (Table 5). The fifth osteopathic care session was scheduled after four weeks.

**Table 5:** Osteopathic diagnosis and treatment of the fourth osteopathic encounter. In the table, an “X” indicates that the reference element for the corresponding cell has been identified, while a “-“ denotes that the reference element has not been found. Body areas for the diagnosis and coding of somatic dysfunction are defined in the ICD-10-CM using codes M99.0-9 (where 0–9 represents the anatomical regions). OMT and OMTh procedures are coded for different body regions and techniques using the codes 7W00-09 (where 00-09 represents the anatomical regions) and X 0-9 Z (where 0–9 indicates the type of technique applied). ICD: International Classification of Diseases; OC: osteopathic care session; OPE: osteopathic palpatory examination; SD: somatic dysfunction; OR: other region, also used to indicate a generalized fascial pattern throughout the body; OMT: osteopathic manipulative treatment; OMTh: osteopathic manipulative therapy; PINS: progressive inhibition of neuromuscular structures technique; MET: muscle energy techniques; MFR: myofascial release; OCF: osteopathy in the cranial field

Osteopathic encounter 4									
OPE - SD								OMT	ICD
ICD	Region	Area	T	A	R	T	Severity	Technique	T. code
M99.00	Head	Occiput	x	-	-	-	Mild	OCF	7W00X4Z
		Temporals	x	-	-	-	Mild	OCF	7W00X4Z
		Temporal m.	-	-	-	-	None	-	-
		Masseter m.	-	-	-	-	None	-	-
M99.01	Cervical	Suboccipital m.	x	-	-	-	Mild	PINS	7W01X9Z
		C1	x	-	-	-	Mild	-	-
		Hyoid	-	-	-	-	None	-	-
		Supra-hyoid m.	-	-	-	-	None	-	-
		Infrahyoid m.	-	-	-	-	None	-	-
M99.02	Thorax	Clavicles	x	-	-	-	Mild	-	-
		T5-T6-T7	x	-	-	x	Moderate	-	-
		Diaphragm m.	-	-	-	-	None	-	-
M99.03	Lumbar	L5-S1	x	-	-	x	Moderate	-	-
M99.04	Sacral	Sacroiliac joints	x	-	-	-	Mild	-	-

Osteopathic encounter 5

No adverse events were reported by the patient after the fourth OMT. The patient resumed sports activities that he had stopped several years earlier (he played as a goalkeeper on a soccer team), and even his social relationships improved. He also returned to university, which he had previously abandoned. Both the neurological consultation and the otolaryngological examination returned negative results. Details of the fifth osteopathic care session are presented in Table 6. The follow-up was scheduled for four weeks later.

**Table 6:** Osteopathic diagnosis and treatment of the fifth osteopathic encounter. In the table, an “X” indicates that the reference element for the corresponding cell has been identified, while a “-“ denotes that the reference element has not been found. Body areas for the diagnosis and coding of somatic dysfunction are defined in the ICD-10-CM using codes M99.0-9 (where 0–9 represents the anatomical regions). OMT and OMTh procedures are coded for different body regions and techniques using the codes 7W00-09 (where 00-09 represents the anatomical regions) and X 0-9 Z (where 0–9 indicates the type of technique applied). ICD: International Classification of Diseases; OC: osteopathic care session; OPE: osteopathic palpatory examination; SD: somatic dysfunction; OR: other region, also used to indicate a generalized fascial pattern throughout the body; OMT: osteopathic manipulative treatment; OMTh: osteopathic manipulative therapy; PINS: progressive inhibition of neuromuscular structures technique; MET: muscle energy techniques; MFR: myofascial release; OCF: osteopathy in the cranial field

Osteopathic encounter 5									
OPE - SD								OMT	ICD
ICD	Region	Area	T	A	R	T	Severity	Technique	T. code
M99.00 M99.01	Head Cervical	Occiput	x	-	-	-	Mild	OCF	7W00X4Z
		Temporal m.	x	-	-	-	Mild	PINS	7W00X9Z
		Masseter m.	x	-	-	-	Mild	PINS	7W01X9Z
		Suboccipital m.	x	-	-	-	Mild	PINS	7W01X9Z
M99.01	Cervical	Visceral c.	x	-	-	-	Mild	MFR	7W01X1Z
		C1	-	-	-	-	None	-	-
		Hyoid	-	-	-	-	None	-	-
		Supra-hyoid m.	-	-	-	-	None	-	-
		Infrahyoid m.	-	-	-	-	None	-	-
M99.02	Thorax	Clavicles	x	-	-	-	Mild	-	-
		T5-T6-T7	-	-	x	-	Mild	-	-
		Diaphragm m.	-	-	x	-	Mild	MFR	7W02X1Z
M99.03	Lumbar	L5-S1	-	-	-	-	None	-	-
M99.04	Sacral	Sacroiliac joints	-	-	-	-	None	-	-

Follow-up and outcomes

**Figure 5:**
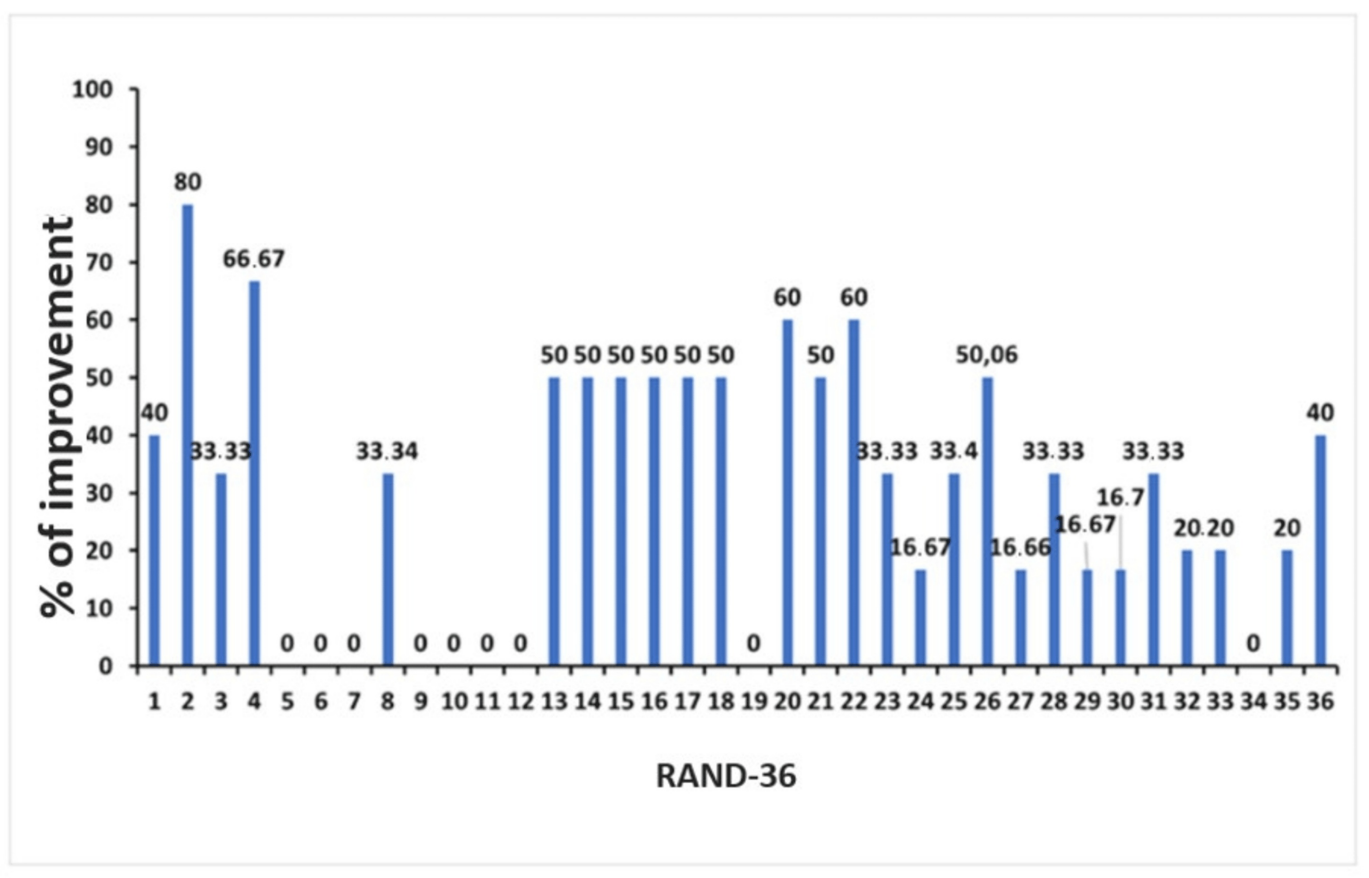
RAND-36 scores at follow-up. Percentage improvement in RAND-36 scores between November 27, 2019 and February 20, 2020. RAND-36: RAND 36-Item Health Survey

No adverse events were reported by the patient after the last OMT and during the follow-up. No OMT was performed at the follow-up visit. The patient completed the final RAND-36 and VAS. The VAS was evaluated before the first OMT and one week after the last OMT. The RAND-36 was administered before the first OMT (November 27, 2019), before the third OMT (December 27, 2019), and one week after the fifth OMT (February 20, 2020). Baseline VAS pain intensity was 9/10, while at the end of the study it had decreased to 2/10. The RAND-36 showed improvements in all domains from baseline to both the one-month and three-month assessments (Figure 5).

## Discussion

The present case report documents the positive effects of OMT in a patient diagnosed with KA, suggesting that collaboration between general practitioners and osteopaths may facilitate earlier recognition and more effective management of symptoms associated with structural anomalies such as KA. The patient presented with a complex symptomatology involving cranio-cervical, neurological, and psychological dimensions, which had not responded to conservative treatments. In addition to the neurologist’s assessment, two factors support the hypothesis that the patient’s clinical presentation was secondary to the presence of KA. First, a complete and structurally robust bony ring has been statistically associated with symptomatic cases [1]. Second, the onset of symptoms at age 16 falls within the developmental “window period” during which the prevalence of symptomatic KA is reported to be higher [1].

Notably, the patient exhibited progressive and multidimensional improvements following a series of five osteopathic sessions, as indicated by reductions in pain intensity (VAS), improvements in quality of life (RAND-36), and a return to daily and academic activities.

Before OMT were applied, the clinical observation of the patient by the osteopath, the osteopath’s clinical observation of the patient underscored the importance of differential diagnosis that included the assessment of somatic dysfunction and consideration of related treatment options, while also integrating complementary care within state-of-the-art pain management practices [22]. In routine clinical practice, patients may seek osteopathic consultation either following an initial evaluation by a general practitioner, as in the present case, or may present directly to the osteopath as their first point of contact for the complaint. In either scenario, strong differential diagnostic skills are essential for any primary-contact healthcare professional. Within the context of this case report, the osteopath conducted a neurological examination that revealed altered sensation and sensory deficits, providing valuable information for the overall clinical assessment and prompting referral to specialists such as an otolaryngologist and a neurologist.

According to the medical literature, when KA is characterized by a complete or partial bony bridge encircling the vertebral artery at the C1 level, patients may present with a range of neurovascular and cervicocephalic symptoms [8]. Although conservative and surgical interventions for KA-related dysfunctions have been reported, the literature lacks documentation on the use of OMT in the management of this condition.

In this case, a person-centered osteopathic care plan was applied, focusing on somatic dysfunction identified through palpatory evaluation and the TART criteria. Despite the structural presence of KA, improvements in functional outcomes were observed without direct treatment of the most severe dysfunction at C1 during the initial session, due to the patient’s apprehension and hypersensitivity. The gradual clinical improvement observed across sessions may reflect the efficacy of individualized and integrative touch-based osteopathic care that emphasized safety, tolerance, and responsiveness to the patient’s embodied experience.

From an osteopathic perspective, and to better contextualize the rationale behind the approach applied in the present case report, particularly in relation to the patient’s chief complaint and the use of OMT, three osteopathic treatment models are worth describing [17]. The first is the standardized approach, in which the osteopath treats one or more predetermined body regions, selecting them based on the pathogenesis of pain, as well as anatomical and physiological considerations. For this reason, OMT is usually targeted at anatomical structures that are either at or near the site of pain, which are often also physiologically involved in the patient’s clinical presentation. The second approach is characterized by a whole-body assessment and treatment, in which the osteopath bases the intervention on findings from the osteopathic structural evaluation, with minimal or no influence from the patient’s chief complaint, anatomical considerations, or the presumed pathogenesis. This approach is also referred to as the “black-box protocol.” The third approach is the semi-standardized model, in which the osteopath partially determines in advance where OMT should be applied, while also considering findings from the clinical evaluation. This approach acknowledges that each patient may present with an individual pattern of somatic dysfunctions or fascial restrictions: each patient receives standardized techniques targeting the region of patient complaint and additional techniques selected according to the findings of the osteopathic assessment. In the present case report, a whole-body approach was adopted. The osteopath treated regions of the body associated with somatic dysfunctions identified during the osteopathic evaluation. Although some techniques were applied to structures anatomically related to C1, this was not based on a predetermined protocol. Rather, the osteopath selected treatment areas according to the severity of findings based on the TART criteria. More specifically, the osteopath, considering both the anatomical and physiological aspects mediated by structures such as the fascial connections of the myodural bridge, continuum linking the spinal dura mater with the suboccipital muscles (rectus capitis posterior minor, rectus capitis posterior major, and obliquus capitis inferior), recognizes that this anatomical relationship may influence head mobility and cerebrospinal fluid dynamics [7]. Furthermore, dysfunctions involving the myodural bridge have been reported to be associated with cervicocephalic headaches and pain syndromes, as well as disturbances in sensorimotor function and postural control [7]. On the other hand, the contemporary concept of tensegrity emphasizes the structural and functional complexity of the neuromusculoskeletal system, aligning with osteopathic principles that promote a holistic understanding of the human body [23]. By considering not only the disease factors but also the patient’s host factors as they relate to an emerging illness state, and by applying holistic reasoning (e.g., osteopathic structure-function models), osteopaths adopt a dynamic approach to organizing osteopathic treatment. This approach is grounded in the identification of the most significant somatic dysfunction within the non-linear continuum of the whole-body fascial network [22]. It also takes into account the available evidence on OMT in patients with headache-related mood disorders [24].

The rationale behind this clinical decision refers to the neurobiological modifications that appear to be induced by OMT [23,25]. These changes have been observed across several body systems, most notably in neurophysiological correlates and musculoskeletal adaptations [23]. Understanding the underlying mechanisms of a patient’s presentation is essential for connecting form and function in OMT, as highlighted by other authors. For example, Chin et al. (2025) [26] reported a case of tension-type headache initially unresponsive to pain medication, which was resolved through OMT and lifestyle modifications. Their approach targeted somatic dysfunction to address the multifactorial mechanisms underlying the patient’s condition, including central and peripheral pain pathways, autonomic regulation, articular and myofascial alterations, personal stress, and environmental stimuli. When developing a treatment sequence, osteopathic practitioners must not only identify the potential causes of dysfunction but also deliver preventive and supportive care to reduce the risk of recurrence.

Currently, within the so-called “tradition-reconceptualization” stream of osteopathic research (see D’Alessandro et al. for a discussion on the tradition-dismissive vs. tradition-reconceptualization debate and a proposed unifying framework [11]), several proposals have been put forward regarding the modernization and evolution of osteopathic practice.

This tradition-respectful reform is unfolding across multiple levels: epistemological, theoretical, person-centered clinical reasoning and decision-making processes, diagnostic palpation criteria for somatic dysfunction, and its related theoretical chemo-physical properties.

**Figure 6:**
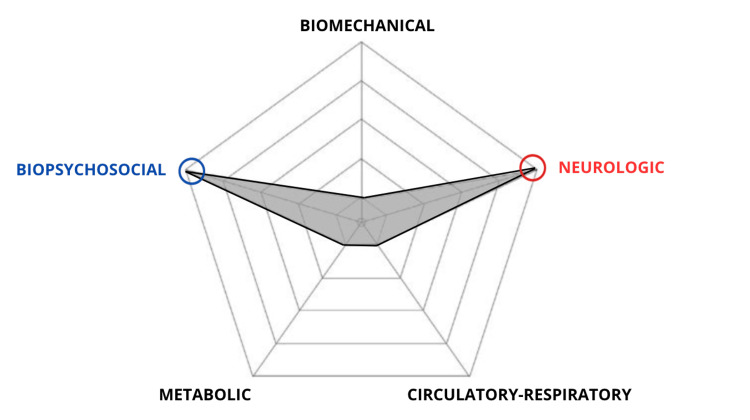
Osteopathic models integration radar plot of the patient. The osteopathic models integration radar plot (modified from Sleszynski et al. [18]) is a tool designed to assist osteopathic practitioners in clinical reasoning, helping them select and integrate various osteopathic models and evidence-based interventions into clinical practice.

The present case report serves as a clear example of the practical application of the “tradition-reconceptualization” perspective in osteopathy. The osteopath adopted a person-centered approach and based the intervention on somatic dysfunctions interpreted through biopsychosocial and neurologic structure/function models (see Figure 6) [22].

It is essential for the osteopathic community to critically reflect on this distinction: the focus of our professional identity should be on somatic dysfunction, rather than on the disease itself.

According to the logic of complex systems, the human body functions in a non-linear manner [11,22]. Therefore, it is essential to adopt a person-tailored osteopathic approach [11,22]. Furthermore, everyone has a unique clinical history of noxious events (e.g., trauma, surgery, tissue degeneration), which can be encoded and retained within the neurological hubs of interoceptive pathways, in line with the so-called Patient-Practitioner-Environment Synchronization framework [11].

We also recommend incorporating differential diagnosis into routine clinical practice for patients presenting with similar symptomatology. In this case report, five OMT sessions had a positive impact on pain and quality of life in a patient presenting with chronic tension-type headache, right-sided neck and trapezius pain (exacerbated by rightward head rotation), dizziness, and occasional hypoacusis and tinnitus in the right ear, in the context of a coexisting KA.

Finally, this case report carries the inherent limitation of being a single-patient observation, with findings that cannot be generalized. Future research should explore the effects of OMT in larger cohorts of patients with radiologically confirmed KA, ideally through controlled clinical trials and the use of multi-modal assessment strategies.

Patient perspective

“Dear Osteopath, I want to sincerely thank you for these sessions, both on my behalf and on behalf of my parents. I truly feel like I’ve been reborn-I have my carefree life back, and thanks to your treatment, I’m even able to play five-a-side football again. I honestly don’t know how to express my gratitude for this incredible gift-it’s been nothing short of a miraculous treatment. I feel like a new person. Thank you so much, and I hope I can return the favor someday.”

## Conclusions

This case report highlights the potential benefits of OMT in a patient with KA, demonstrating significant improvements in pain, quality of life, and daily functionality. The patient’s complex presentation, involving cranio-cervical, neurological, and psychological components, had not responded to previous recommended treatments; however, the individualized OMT approach led to positive outcomes, also underlined by the patient’s perspective. Notably, the success of OMT in this case emphasizes the importance of a collaborative approach between general practitioners and osteopaths, which could facilitate earlier detection and more effective management of symptoms associated with structural anomalies such as KA. The integration of differential diagnosis, comprehensive clinical evaluation, and patient-centered care further contributed to the patient’s progress. While the findings of this case report are promising, they are limited by the single-patient design. Future research involving larger cohorts of patients with KA, including controlled trials and multi-modal assessment strategies, is essential to validate these results and expand our understanding of OMT’s efficacy in managing KA-related symptoms. As osteopathic practice evolves, it is crucial to adopt a biopsychosocial, patient-centered model that acknowledges the complex, non-linear nature of the body’s response to dysfunction and treatment.

## References

[1] Haładaj R, Haładaj R, Haładaj N, Varga I (2025). Bony bridges and selected variations of the first cervical vertebra(atlas): a brief encyclopedic overview with comments on clinical and neurological implications. Transl Res Anat.

[2] Buna M, Coghlan W, deGruchy M, Williams D, Zmiywsky O (1984). Ponticles of the atlas: a review and clinical perspective. J Manipulative Physiol Ther.

[3] Sekerci AE, Soylu E, Arikan MP, Ozcan G, Amuk M, Kocoglu F (2015). Prevalence and morphologic characteristics of ponticulus posticus: analysis using cone-beam computed tomography. J Chiropr Med.

[4] Elliott RE, Tanweer O (2014). The prevalence of the ponticulus posticus (arcuate foramen) and its importance in the Goel-Harms procedure: meta-analysis and review of the literature. World Neurosurg.

[5] Pękala PA, Henry BM, Pękala JR (2017). Prevalence of foramen arcuale and its clinical significance: a meta-analysis of 55,985 subjects. J Neurosurg Spine.

[6] Gibelli D, Cappella A, Cerutti E, Spagnoli L, Dolci C, Sforza C (2016). Prevalence of ponticulus posticus in a Northern Italian orthodontic population: a lateral cephalometric study. Surg Radiol Anat.

[7] Sung YH (2022). Suboccipital muscles, forward head posture, and cervicogenic dizziness. Medicina (Kaunas).

[8] Lvov I, Lukianchikov V, Grin A, Sytnik A, Polunina N, Krylov V (2017). Minimally invasive surgical treatment for Kimmerle anomaly. J Craniovertebr Junction Spine.

[9] Kuć J, Szarejko KD, Aleksandrowicz K, Gołębiewska M (2021). The role of soft tissue mobilization in reducing orofacial and general complaints in a patient with Kimmerle anomaly and temporomandibular joint disorder: a case report. Cranio.

[10] Riley DS, Barber MS, Kienle GS (2017). CARE guidelines for case reports: explanation and elaboration document. J Clin Epidemiol.

[11] D’Alessandro G, Lunghi C, Consorti G (2025). Tradition-dismissive vs. tradition reconceptualization approaches in musculoskeletal care: the example of osteopathic care. Appl Sci.

[12] Swift A (2015). The importance of assessing pain in adults. Nurs Times.

[13] Fava GA, McEwen BS, Guidi J, Gostoli S, Offidani E, Sonino N (2019). Clinical characterization of allostatic overload. Psychoneuroendocrinology.

[14] Walton DM, Elliott JM (2018). A new clinical model for facilitating the development of pattern recognition skills in clinical pain assessment. Musculoskelet Sci Pract.

[15] Agrawal Y, Carey JP, Hoffman HJ, Sklare DA, Schubert MC (2011). The modified Romberg Balance Test: normative data in U.S. adults. Otol Neurotol.

[16] Hasvik E, Haugen AJ, Grøvle L (2021). Pinprick and light touch are adequate to establish sensory dysfunction in patients with lumbar radicular pain and disc herniation. Clin Orthop Relat Res.

[17] Tramontano M, Tamburella F, Dal Farra F (2021). International overview of somatic dysfunction assessment and treatment in osteopathic research: a scoping review. Healthcare (Basel).

[18] Sleszynski SL, Glonek T, Kuchera WA (1999). Standardized medical record: a new outpatient osteopathic SOAP note form: validation of a standardized office form against physician's progress notes. J Am Osteopath Assoc.

[19] Delgado DA, Lambert BS, Boutris N, McCulloch PC, Robbins AB, Moreno MR, Harris JD (2018). Validation of digital Visual Analog Scale pain scoring with a traditional paper-based Visual Analog Scale in adults. J Am Acad Orthop Surg Glob Res Rev.

[20] García-Sánchez E, Santamaría-Peláez M, Benito Figuerola E (2024). Comparison of SF-36 and RAND-36 in cardiovascular diseases: a reliability study. J Clin Med.

[21] Apolone G, Mosconi P (1998). The Italian SF-36 Health Survey: translation, validation and norming. J Clin Epidemiol.

[22] Castagna C, Consorti G, Turinetto M, Lunghi C (2021). Osteopathic models integration radar plot: a proposed framework for osteopathic diagnostic clinical reasoning. J Chiropr Humanit.

[23] Dal Farra F, Bergna A, Lunghi C, Bruini I, Galli M, Vismara L, Tramontano M (2024). Reported biological effects following osteopathic manipulative treatment: a comprehensive mapping review. Complement Ther Med.

[24] D'Ippolito M, Tramontano M, Buzzi MG (2017). Effects of osteopathic manipulative therapy on pain and mood disorders in patients with high-frequency migraine. J Am Osteopath Assoc.

[25] Ponzo V, Cinnera AM, Mommo F, Caltagirone C, Koch G, Tramontano M (2018). Osteopathic manipulative therapy potentiates motor cortical plasticity. J Am Osteopath Assoc.

[26] Chin J, Qiu W, Lomiguen CM, Volokitin M (2020). Osteopathic manipulative treatment in tension headaches. Cureus.

